# Predictors of faster virological suppression in early treated infants with perinatal HIV from Europe and Thailand

**DOI:** 10.1097/QAD.0000000000002172

**Published:** 2019-02-07

**Keywords:** early combination antiretroviral therapy, infants, perinatal HIV, predictors, virological suppression

## Abstract

**Objective:**

To identify predictors of faster time to virological suppression among infants starting combination antiretroviral therapy (cART) early in infancy.

**Design:**

Cohort study of infants from Europe and Thailand included in studies participating in the European Pregnancy and Paediatric HIV Cohort Collaboration.

**Methods:**

Infants with perinatal HIV starting cART aged less than 6 months with at least 1 viral load measurement within 15 months of cART initiation were included. Multivariable interval-censored flexible parametric proportional hazards models were used to assess predictors of faster virological suppression, with timing of suppression assumed to lie in the interval between last viral load at least 400 and first viral load less than 400 copies/ml.

**Results:**

Of 420 infants, 59% were female and 56% from Central/Western Europe, 26% United Kingdom/Ireland, 15% Eastern Europe and 3% Thailand; 46 and 54% started a boosted protease inhibitor-based or nonnucleoside reverse transcriptase inhibitor-based regimen, respectively. At cART initiation, the median age, CD4^+^% and viral load were 2.9 [interquartile range (IQR): 1.4–4.1] months, 34 (IQR: 24–45)% and 5.5 (IQR: 4.5–6.0) log_10_ copies/ml, respectively. Overall, an estimated 89% (95% confidence interval: 86–92%) achieved virological suppression within 12 months of cART start. In multivariable analysis, younger age [adjusted hazard ratio (aHR): 0.84 per month older; *P* < 0.001], higher CD4^+^% (aHR: 1.11 per 10% higher; *P* = 0.010) and lower log_10_ viral load (aHR: 0.85 per log_10_ higher; *P* < 0.001) at cART initiation independently predicted faster virological suppression.

**Conclusion:**

We observed a significant independent effect of age at cART initiation, even within a narrow 6 months window from birth. These findings support the earliest feasible cART initiation in infants and suggest that early therapy influences key virological and immunological parameters that could have important consequences for long-term health.

## Abstract 2

Supplemental Digital Content is available in the text

## Introduction

The clinical benefits of starting combination antiretroviral therapy (cART) early in HIV-infected infants have been demonstrated in several observational studies and trials. The Children with HIV Early Antiretroviral Therapy (CHER) trial showed that starting cART before 12 weeks of age markedly reduces mortality and morbidity in HIV-infected infants [1,2]. These findings were confirmed in the European Infant Collaboration (EIC) observational study [3] and the French perinatal cohort [4]. Several other studies have shown that early cART initiation controls proviral replication [5], limits establishment of latent HIV reservoirs [6–8], preserves immune function [9] and contributes to long-term viral suppression [10,11]. Rare case reports have further highlighted the beneficial effect of early cART in sustaining HIV remission in children who subsequently discontinue cART [12–15].

Several studies investigating virological outcomes have also demonstrated that early cART improves initial viral control in infants, although results have been variable. For instance, an Italian study reported that infants starting cART at less than 6 months of age had better virological responses compared with those starting after 6 months of age over all follow-up time-points [16]. A subgroup analysis of data from the EIC cohort showed that early therapy within 3 months of life was associated with a faster control of viral replication [11]. Similarly, a trial evaluating safety and tolerability of three cART regimens in the United States and Puerto Rico showed that early therapy in the first 3 months of life was associated with improved long-term viral suppression [17]. However, in contrast, a recent South African study reported inconsistent benefits of early cART on timing of virological suppression in infants starting cART less than 6 months of age [18]. In addition, few studies have examined factors associated with time to virological suppression in infants starting cART before 6 months of age; most studies have evaluated children initiating cART during the first year of life [19–21] or at older ages [22] and results have been variable.

Therefore, the aim of our study was to investigate predictors of faster virological suppression, focusing on infants with perinatal HIV initiating standard cART within the first 6 months of life living in Europe and Thailand and included in studies participating in the European Pregnancy and Paediatric HIV Cohort Collaboration (EPPICC).

## Methods

### Study inclusion criteria

Data from the EPPICC Paediatric merger 2014 (*n* = 3953) were used, as described previously [23,24]. In brief, the EPPICC merged individual patient data on routine demographic, clinical, laboratory and treatment-related variables from 19 observational cohorts across 17 countries prepared according to a standardised data specification. The inclusion criteria for this analysis were infants with perinatal HIV and aged less than 6 months at start of standard cART, defined as boosted protease inhibitor (bPI) or nonnucleoside reverse transcriptase inhibitor (NNRTI) and two or more NRTIs, with a baseline viral load measure at cART initiation and at least one viral load within the 15 months following initiation.

### Statistical analysis

The endpoint for this analysis was virological suppression. The exact time of viral suppression could not be observed and it was only known to have occurred at some point in an interval of time. Therefore, interval-censored methods for analysing survival data were required to accurately estimate the distribution of time of the event (i.e. virological suppression). Owing to the nature of our observational cohort study, there were natural differences in follow-up visit schedules across regions resulting in subsequent differences in gap between consecutive viral load measurements by region. As a result, interval-censored methods were also required to estimate an unbiased effect of region. In our main analysis, the time of virological suppression was assumed to have occurred in the interval between last viral load at least 400 and first viral load less than 400 copies/ml [referred as ‘virological suppression (Interval)’ thereafter]. Time from cART initiation to virological suppression was analysed using time-to-event methods, censoring follow-up at the earliest of last viral load measurement before more than 15-month gap in measurements or last viral load measurement. The cumulative probability of infants achieving virological suppression over time from cART initiation was estimated using interval-censored flexible parametric survival models. In sensitivity analyses, cumulative probabilities were also estimated using Kaplan–Meier methods and nonparametric maximum likelihood estimate (MLE) for interval-censored data, as implemented in the R package *interval* (R Core Team (2017). R: A language and environment ßor statistical computing. R Foundation for Statistical Üomputing, Vienna, Austria. URL https://www.R-project.org/) [25].

Univariable and multivariable interval-censored flexible parametric proportional hazards survival models were used to identify predictors of faster virological suppression. These models were chosen as the main analysis to allow for analysis of interval censored data, where virological suppression was assumed to occur in the interval between last viral load at least 400 and first viral load less than 400 copies/ml. These models further extend standard parametric models using restricted cubic splines rather than linear functions for the underlying log cumulative hazard, as implemented in Stata stpm function [26]. Akaike information criteria (AIC) was used to identify the best-fitting model (lowest AIC), testing 1–6 degrees of freedom of the underlying spline for the log cumulative hazard. Predictors identified from univariable models with *P* less than 0.10 met criteria for inclusion into multivariable model, along with those identified *a priori* (geographical region and initial cART regimen). Backward stepwise elimination (exit probability *P* = 0.05) was applied to reach the final multivariable model. The functional form of significant predictors was explored using regression splines.

We evaluated the following factors: age at cART initiation, baseline viral load, CD4^+^% and cell count, sex, ethnicity, initial cART regimen (bPI or NNRTI and two or more NRTI), infant antiretroviral prevention of mother-to-child transmission (PMTCT) prophylaxis regimen given within 4 weeks of birth, maternal antiretroviral PMTCT regimen used in the prenatal and delivery period, birth abroad (whether infants were born in the same country in which they were enrolled for HIV care), year of birth, year of cART initiation, Centers for Disease Control and Prevention (CDC) C event by cART initiation and geographical region. Geographical region of cohort was categorised *a priori*, as described previously [24]: Eastern Europe (Russia and Ukraine), Central and Western Europe (Belgium, Germany, Italy, Netherlands, Poland, Portugal, Romania, Spain, Sweden, Switzerland, France and Greece), United Kingdom/Ireland and Thailand. Missing values for baseline viral load (18%) and CD4^+^% (22%) were multiply imputed by chained equations (20 cycles), on the complete EPPICC dataset to avoid imputation bias. Imputation diagnostics was performed by adjusting number of imputations to minimize proportion of total sampling variance that is due to missing data.

Sensitivity analyses were carried out using both multiply imputed and nonimputed data (complete case analysis) and by considering two additional definitions of time of virological suppression: first, Midpoint between last viral load at least 400 and first viral load less than 400 copies/ml [referred as ‘virological suppression (Midpoint)’]; second, date viral load less than 400 copies/ml [referred as ‘virological suppression (Observed)’]. The proportional hazards assumption for each predictor was tested and accounted for by including predictor × time (_*t*) interaction term in the multivariable model, where applicable. To further confirm results, Cox proportional hazards models and interval-censored parametric survival models with Weibull distribution were also fitted. Stata version 15.1 (Stata Corporation, College Station, Texas, USA) was used for all analyses, unless otherwise stated.

## Results

### Patient characteristics

**Fig. 1:**
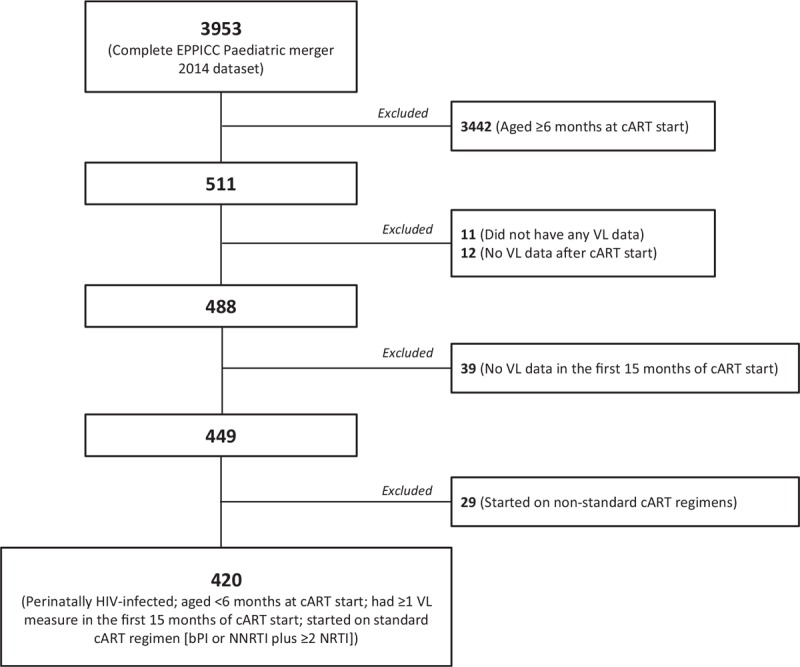
Patient inclusion flow chart.

Of the 3953 children included in the dataset, 420 (11%) infants met the study inclusion criteria of being perinatally HIV-infected, aged less than 6 months at cART initiation with at least 1 viral load measurement within 15 months of cART start (1998–2013) (Fig. 1). Of these, 59% were female, 35% were white and 29% were of black ethnicity (Table 1). Most (90%) were born in the same country in which they were enrolled for HIV care and the vast majority (94%) were born after the year 2000. In total, 56% were from Central/Western Europe, 26% from United Kingdom/Ireland, 15% from Eastern Europe and 3% from Thailand. Among infants that had PMTCT prophylaxis data available, over half (59%) received PMTCT and 44% received maternal PMTCT, and 16% had a CDC C event by the time of cART initiation.

This figure shows patient inclusion selection from the complete European Pregnancy and Paediatric HIV Cohort Collaboration Paediatric merger 2014 dataset.

**Table 1:** Baseline characteristics.

*N* = 420	Median (IQR) or *N* (%)
Sociodemographic characteristics, *N*^a^	
Sex	
Male	173 (41%)
Female	247 (59%)
Ethnicity^b^	
White	146 (35%)
Black	121 (29%)
Other	19 (5%)
Unknown	134 (32%)
Geographical region	
UK/Ireland	109 (26%)
Thailand	14 (3%)
Eastern Europe	61 (15%)
Central and Western Europe	236 (56%)
Birth abroad, *N* = 415	
No	374 (90%)
Yes	41 (10%)
Year of birth	
<2000	24 (6%)
≥2000	396 (94%)
HIV-related parameters	
Baseline	
CD4^+^%, *N* = 329	34 (24–45)
CD4^+^ (cells/μl), *N* = 340	1781 (995–2644)
Viral load (copies/ml), *N* = 344	314 116 (34 324–1000 000)
Log_10_ viral load (copies/ml), *N* = 344	5.5 (4.5–6.0)
CDC C event by cART initiation	
No	353 (84%)
Yes	67 (16%)
ART-related characteristics	
Age at cART initiation (months)	2.9 (1.4–4.1)
Year of cART initiation	
1998–<2004	105 (25%)
2004–<2008	133 (32%)
≥2008	182 (43%)
Initial cART regimen	
bPI + ≥2 NRTI	194 (46%)
NNRTI + 2 NRTI	150 (36%)
NNRTI + 3 NRTI	76 (18%)
Maternal PMTCT^c^, *N* = 326	
No	181 (56%)
Yes	145 (44%)
If yes, the most potent PMTCT regimen used in the prenatal and delivery period, *N* = 145	
PMTCT given but regimen unknown	10 (7%)
Mono or dual therapy including an NNRTI	20 (14%)
Triple therapy including an NNRTI	15 (10%)
Triple therapy not including an NNRTI	70 (48%)
Other	30 (21%)
Infant PMTCT^d^, *N* = 332	
No	135 (41%)
Yes	197 (59%)
If yes, the most potent PMTCT regimen given within 4 weeks of birth, *N* = 197	
PMTCT given but regimen unknown	15 (7.6%)
Mono or dual therapy including an NNRTI	56 (28%)
Triple therapy including an NNRTI	41 (21%)
Triple therapy not including an NNRTI	7 (3.6%)
Other	78 (40%)

bPI, boosted protease inhibitor – lopinavir (LPV); cART, combination antiretroviral therapy; CDC, Centers for Disease Control and Prevention; IQR, interquartile range; NNRTI, nonnucleoside reverse transcriptase inhibitors; nucleoside reverse transcriptase inhibitors; PMTCT, prevention of mother-to-child transmission; VL, viral load.

^a^Numbers shown if data were available in less than 420 infants.

^b^Ethnicity listed as a separate category if more than 20 per category, otherwise these are combined into the ‘Other’ category.

^c^Maternal PMTCT regimen used in the prenatal and delivery period.

^d^Infant PMTCT regimen given within 4 weeks of birth.

Median age at cART initiation was 2.9 (interquartile range: 1.4–4.1) months, with 43% initiating cART after the year 2008. Forty-six percent of infants started on a bPI (lopinavir) based regimen, and 54% on a NNRTI (nevirapine) based regimen. Median CD4^+^%, CD4^+^ cell count and viral load at cART initiation were 34 (24–45)%, 1781 (995–2644) cells/μl and 5.5 (4.5–6.0) log_10_ copies/ml, respectively (Table 1). Of the 420 infants included, 17 (4%) had one viral load after cART start. The remaining 403 (96%) had at least two viral loads. The median and mean number of viral load measurements after cART start was 15 and 19 per infant, respectively. Median duration of follow-up after cART initiation was 5.2 (2.1–8.8) years. Median gap between consecutive viral load measurements was 9 (5–13) weeks and this varied by geographical region, ranging from 7 weeks in the United Kingdom/Ireland, 9 weeks in Central/Western Europe, 17 weeks in Thailand to 18 weeks in Eastern Europe. Baseline viral load was missing in 76 infants (18%). These missing values were multiply imputed for analysis. The numbers missing baseline viral load by age at cART start bracket were similar. The proportions missing versus nonmissing baseline viral load were also not significantly different by age bracket (*P* = 0.795) (Supplementary Appendix 1).

### Cumulative probability of virological suppression

Overall, the cumulative probability of achieving virological suppression (Interval) by 12 months after cART initiation was estimated at 89% [95% confidence interval (CI): 86–92]%, using the interval-censored flexible parametric survival model (Supplementary Fig. 1). In sensitivity analyses, the cumulative probabilities estimated by the nonparametric MLE method were very similar to those obtained using the parametric survival model. The probabilities of virological suppression (Midpoint) and (Observed) by 12 months were estimated (Kaplan–Meier) at 84% (80–87)% and 77% (73–81)%, respectively (Supplementary Fig. 1).

### Baseline predictors of virological suppression

**Fig. 2:**
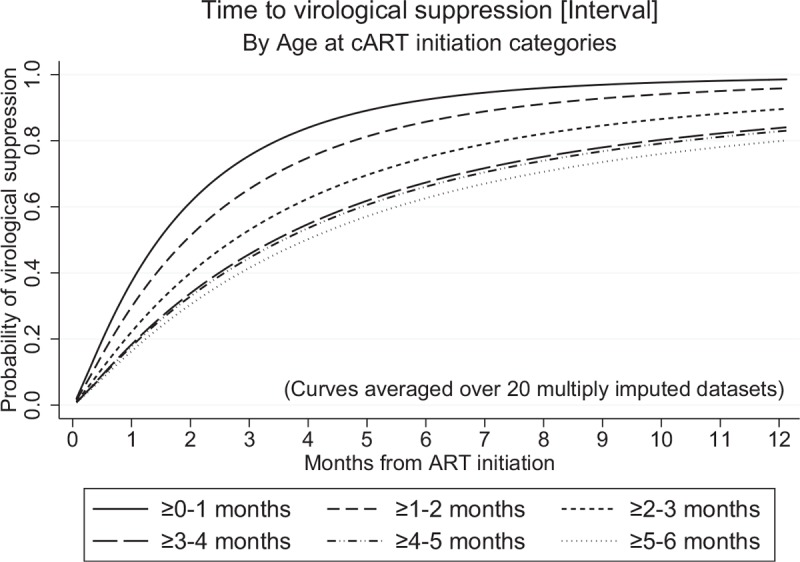
Effect of age at combination antiretroviral therapy initiation on time to virological suppression (Interval).

Results of interval-censored univariable and multivariable analyses using multiply imputed data are shown in Table 2. In multivariable analysis, independent predictors of faster virological suppression (Interval) were younger age at cART initiation [adjusted hazard ratio (aHR): 0.84 (95% CI: 0.78–0.91) per month older; *P* < 0.001], higher baseline CD4^+^% [aHR: 1.11 (95% CI: 1.03–1.20) per 10% higher; *P* = 0.010] and lower baseline log_10_ viral load [aHR: 0.85 (95% CI: 0.78–0.93) per log_10_ higher; *P* < 0.001], adjusting for initial cART regimen and geographical region. Figure 2 illustrates the significant effect of age at cART initiation on time to virological suppression. Of note, in univariable analysis, higher CD4^+^ cell count also predicted faster virological suppression. However, due to multicollinearity with CD4^+^%, CD4^+^ cell count was not included in the final multivariable model. CD4^+^% was included as it is a more stable measurement in children aged less than 5 years [21,27]. There was no significant effect of any of the remaining factors examined on time to virological suppression (Table 2).

**Table 2:** Univariable and multivariable predictors of virological suppression (Interval).

	Univariable model			Multivariable model^*^		
Predictors	HR	(95% CI)	*P* value	aHR	(95% CI)	*P* value
**At cART initiation**						
Age (per month older)	0.83	(0.77–0.88)	**<0.001**	0.84	(0.78–0.91)	**<0.001**
CD4^+^% (per 10% higher)	1.16	(1.08–1.24)	**<0.001**	1.11	(1.03–1.20)	**0.010**
CD4^+^ cell count (per 500 cell higher)	1.13	(1.07–1.18)	**<0.001**	–	–	–
Viral load (per log_10_ higher)	0.80	(0.74–0.87)	**<0.001**	0.85	(0.78–0.93)	**<0.001**
Sex (Ref: male)						
Female	1.03	(0.83–1.27)	0.810			
Infant PMTCT (Ref: no)						
Yes	1.16	(0.91–1.47)	0.221			
Maternal PMTCT (Ref: no)						
Yes	1.19	(0.94–1.51)	0.145			
Birth abroad (Ref: no)						
Yes	0.99	(0.70–1.40)	0.968			
Year of birth (Ref: <2000)						
≥2000	1.44	(0.93–2.24)	0.106	–	–	–
CDC C event by cART initiation (Ref: no)						
Yes	0.81	(0.61–1.08)	0.151			
Year of cART initiation (Ref: 1998–<2004)						
2004–<2008	0.95	(0.73–1.24)	0.695	–	–	–
≥2008	1.27	(0.98–1.65)	0.073	–	–	–
Initial cART regimen^a^ (Ref: bPI + NRTI)						
NNRTI + 2 NRTI	0.95	(0.75–1.21)	0.696	0.83	(0.64–1.07)	0.156
NNRTI + 3 NRTI	1.04	(0.78–1.39)	0.780	0.93	(0.63–1.39)	0.740
Ethnicity (Ref: Black)						
White	0.86	(0.66–1.11)	0.246			
Other	1.11	(0.66–1.87)	0.700			
Unknown	0.94	(0.72–1.22)	0.645			
Geographical region^a^ (Ref: Central/Western Europe)						
UK/Ireland	1.08	(0.85–1.37)	0.540	1.29	(0.93–1.80)	0.131
Thailand	0.97	(0.54–1.75)	0.921	1.77	(0.92–3.39)	0.085
Eastern Europe	0.88	(0.64–1.21)	0.434	1.13	(0.79–1.62)	0.505

In bold, *P* less than 0.05; bPI, boosted protease inhibitor – lopinavir (LPV); CDC, Centers for Disease Control and Prevention; NRTI, nucleoside reverse transcriptase inhibitors; NNRTI, nonnucleoside reverse transcriptase inhibitor – nevirapine (NVP); baseline VL was defined as closest measurement within 6 months before and 1 week after cART initiation and was expressed in copies/ml; baseline CD4^+^% and cell count were defined as closest measurements within 6 months before and 1 month after cART initiation. In all cases, the closest pre-cART measurement was taken, if available. *Note:* In univariable analysis, higher CD4^+^ cell count also predicted faster virological suppression. However, due to multicollinearity with CD4^+^%, CD4^+^ cell count was not included in the final multivariable model. CD4^+^% was included as it is a more stable measurement in children aged less than 5 years than CD4^+^ cell count [21,27]. 95% CI, 95% confidence interval; aHR, hazard ratio adjusted for the other factors included the multivariable model; cART, combination antiretroviral therapy; HR, hazard ratio; PMTCT, prevention of mother-to-child transmission.

^*^Criteria for inclusion into the multivariable model: univariable model *P* less than 0.10, along with those identified *a priori*^a^ (geographical region and initial cART regimen).

This figure shows the estimated cumulative probabilities of achieving virological suppression (Interval) by age at combination antiretroviral therapy initiation during the first 12 months of antiretroviral therapy. Probabilities were estimated from the flexible parametric survival model using multiply imputed data. Curves were averaged over 20 multiply imputed datasets.

### Sensitivity analyses of predictors of virological suppression

Restricted to the complete case analysis, the same factors (age at cART initiation, baseline CD4^+^% and viral load) remained significantly associated with faster virological suppression (Interval) (Supplementary Table 1). Regression spline analysis further revealed a linear relationship of significant predictors with the outcome (Supplementary Fig. 2). Results from multivariable analyses of time to virological suppression (Midpoint) and (Observed) were similar, both with and without multiple imputation (Supplementary Tables 1 and 2). In some sensitivity analyses, initial cART regimen and/or geographical region were found to violate the proportional hazards assumption (i.e. interact with analysis time) hence, corresponding interaction terms were included in the relevant final multivariable models to account for this effect. Additional univariable and multivariable analyses using Cox proportional hazards models and interval-censored parametric survival models with Weibull distribution further confirmed the main findings (data not shown).

## Discussion

To our knowledge, this is the largest study reported to date exploring predictors of faster virological response in infants with perinatal HIV initiating cART within 6 months of life. Most infants achieved virological suppression within 12 months of starting cART. This is the first study to show that even amongst infants initiating therapy within 6 months of life, earlier cART initiation predicted faster virological suppression. Higher baseline CD4^+^% and lower log_10_ viral load were also independent predictors of faster virological suppression.

Our findings are consistent with the general conclusions from smaller studies that have identified early treatment as a determinant of faster virological suppression. The European EIC study reported faster suppression in infants starting cART earlier (<3 months of age) compared with later treated infants [11]. In another study (*n* = 128) investigating older ART naïve African children aged 1.7–13.5 years, children younger than 8 years had increased probability of attaining virological suppression compared with older children [28]. Other studies conducted in the United States/Puerto Rico (*n* = 52), Italy (*n* = 133) and South Africa (*n* = 1748; total of five cohorts), revealed that early therapy was a significant predictor of long-term viral suppression in children starting cART at less than 3 months (versus ≥3 months of age) [17] and less than 6 months (versus ≥6 months of age) [16,18]. Although determining the onset of infection in adults is problematic, recent studies specifically designed to capture this information, have also identified early ART during primary infection as a key factor for faster viral suppression in adults [29].

The estimated cumulative probability of achieving virological suppression (Interval), (Midpoint) and (Observed) was 89, 84 and 77%, respectively. This is consistent with previous findings despite the variability in definitions of suppression. In the CHER trial (*n* = 377) conducted in South Africa, the proportion of infants with viral load less than 400 copies/ml by 12 months post-cART initiation was 77% [30]. Similar findings were reported by the EIC study among 139 infants starting cART before 3 months of age [11]. In two studies conducted in Kenya (*n* = 121) and Mozambique (*n* = 119), the proportion of children suppressed to less than 1000 copies/ml was 75% [31] and 77% [32], respectively, by 12 months after cART start. Similarly, in a Ugandan study (*n* = 91), the probability of suppression to less than 400 copies/ml was 83.1% in infants starting cART less than 12 months of age [33]. Further evidence that with early intervention high levels of suppression can be achieved comes from a pilot trial of infants randomised to receive immediate or deferred four-drug (three-class) antiretroviral therapy (*n* = 63) [19] at a median age of 28 days (intrauterine-infected) and 55 days (intrapartum-infected). The proportions attaining virological suppression to less than 400 copies/ml and less than 50 copies/ml were higher at 100% and 94%, respectively, 12 months post-cART.

The reasons for the critical timing of cART initiation on virological suppression are unclear. The relationship with lower baseline viral load has been observed before in various settings and at different ages [17,19,21,22,34]. There are sound virological and immunological reasons why an individual with lower levels of circulating virus would suppress more rapidly. The viral decay following initiation of ART occurs in phases. Firstly, the initial decline signifies early loss of short-lived virally productive cells. This is followed by the slower loss of longer lived but productive cells. Finally, there is the much slower loss of latently infected cells [35]. In the context of our findings, the implication would be that earlier treatment may target the short-lived cells, which could have important implications for limiting viral reservoirs. This in turn may also provide optimal opportunities for adjunctive therapies as part of the cure agenda [36].

As well as baseline viral load, faster virological suppression was also associated with a higher baseline CD4^+^% (also CD4^+^ cell count). This association has been seen in multiple studies [19,20,34] in infants, children, pregnant women and adults. Although it is tempting to link the lower viral load with the higher CD4^+^%, they were actually independent predictors of viral suppression, indicating an immunological basis for this finding. Our previous work has shown how critical early treatment is for preserving CD4^+^ cell counts [37]. In the CHER study, even though early treatment arrested CD4^+^ decline, it did not fully restore levels to those seen in HIV-uninfected children [37]. When therapy was stopped as part of planned treatment interruption, there was a rapid decline in CD4^+^ T-cells, which on retreatment returned to levels observed before interruption. This indicates that baseline CD4^+^ levels provides insight into CD4^+^ cell homeostasis, with individuals with higher CD4^+^ having a greater proportion of recent thymic emigrants, which are relatively resistant to HIV infection [37]. The combination of early treatment, low viral load and high baseline CD4^+^ cells is therefore desirable for many reasons including faster immune reconstitution [31], limiting viral reservoir seeding, preserving age appropriate CD4^+^ cell homeostasis and providing opportunities for ‘HIV cure’ [38]. Infants with these characteristics may represent the target population in which to investigate therapeutic vaccines, with the ultimate goal of achieving ART-free HIV remission. Therapeutic vaccines are an integral part of the HIV cure agenda and an increasing global health priority [38].

Apart from age at cART initiation, baseline CD4^+^% and viral load, none of the remaining factors examined predicted faster virological suppression in our final multivariable analysis. A few other studies in Europe have, however, reported significant associations with initial cART regimen [21] and calendar year [22] in older children starting cART less than 18 years of age. On the other hand, in an Ugandan study, none of the baseline factors investigated (age, sex, CD4^+^%, WHO stage, cART regimen, weight-for-age or height-for-age *z*-scores) were found to predict virological suppression to viral load less than 400 copies/ml [33]. Although there was no significant effect of infant and maternal PMTCT prophylaxis on time to virological suppression, the potential effect of PMTCT on suppression has recently been demonstrated [39].

Our study had limitations. It was a subgroup analysis of pooled observational cohort data, hence potential effects of selection bias and unmeasured confounders cannot be ruled out. Data on exact timing of HIV infection and antiviral treatment adherence were not available and could not be investigated. Although year of cART initiation was not associated with time to virological suppression, our data date back to 1998. Given the changes in treatment guidelines over time across countries, all analyses were adjusted for initial cART regimen and geographical region despite their lack of association with the outcome. Longitudinal investigation of long-term viral suppression in early treated infants is also of interest, but was outside the scope of this article. Finally, although our study supports the earliest feasible cART initiation in infants, there are still challenges that need to be addressed before infants can indeed benefit from very early therapy. These challenges relate to difficulties in scaling up birth testing in low and middle income countries with the highest burden [40–42].

In conclusion, we showed that effective treatment response was achieved in the majority of infants initiating cART within 6 months of life across Europe and Thailand. We identified the conditions needed to attain faster virological suppression in these infants. We demonstrated that even amongst early treated infants, earlier cART initiation, higher baseline CD4^+^% and lower baseline viral load independently predicted faster virological suppression. These results provide additional support for earlier cART initiation in infants with perinatal HIV and indicate that early treatment influences key virological and immunological parameters that could have important consequences for long-term health.

## Acknowledgements

We thank all the patients for their participation in these cohorts, and the staff members who cared for them.

**Writing Group** Consisting of Project Team first (ordered alphabetically by name except for the first and last authors for each study team), and also other Writing Group members (ordered alphabetically by cohort name):

*EPPICC/EPIICAL Project Team:* Man K. Chan (EPIICAL statistician), Ruth Goodall (EPPICC senior statistician), Ali Judd (EPPICC colead), Nigel Klein [Collaborative HIV Paediatric Study (CHIPS), UK and Ireland], Elena Chiappini (Italian Register for HIV Infection in Children, Italy), Thomas Klimkait (Swiss Mother and Child HIV Cohort Study, Switzerland), Nicole Ngo-Giang-Huong [Thailand Program for HIV Prevention and Treatment (PHPT), Thailand], Paolo Palma (EPIICAL colead), Paolo Rossi (EPIICAL scientific coordinator), Claire Thorne (EPPICC colead), Anna Turkova [Paediatric European Network for the Treatment of AIDS (PENTA), Italy], Paola Zangari (EPIICAL scientific coordination), Pablo Rojo (EPIICAL colead), Abdel G. A. Babiker (EPIICAL senior statistician).

*Other Writing Group members:* Pieter L. Fraaij, Dasja Pajkrt (ATHENA paediatric cohort, Netherlands); Laura Marques (Centro Hospitalar do Porto, Portugal); Intira J. Collins, Diana M. Gibb [Collaborative HIV Paediatric Study (CHIPS), UK & Ireland]; Maria I. González-Tome, Jose T. Ramos, María L. Navarro (Madrid and CoRISPE cohort, Spain); Antoni Noguera-Julian (CoRISPE-cat cohort, Spain); Josiane Warszawski (French Perinatal Cohort Study, France); Christoph Königs (German Pediatric and Adolescent HIV cohort, Germany); Vana Spoulou (Greece Cohort, Greece); Filipa Prata (Hospital de Santa Maria/CHLN, Lisbon, Portugal); Tessa Goetghebuer (Hospital St Pierre paediatric cohort, Belgium); Luisa Galli (Italian Register for HIV infection in children, Italy); Lars Naver (Karolinska Institutet and University Hospital, Stockholm, Sweden); Carlo Giaquinto [Paediatric European Network for the Treatment of AIDS (PENTA), Italy]; Magdalena Marczynska (Polish paediatric cohort, Poland); Liubov Okhonskaia (Republican Hospital of Infectious Diseases, St Petersburg, Russia); Ruslan Malyuta, Alla Volokha (Ukraine Paediatric HIV Cohort Study, Odessa, Ukraine); Luminita Ene (‘Victor Babes’ Hospital Cohort, Romania).

*The EPIICAL Consortium study team:* Nigel Klein, Diana Gibb, Sarah Watters, Man Chan, Laura McCoy, Abdel Babiker (University College London, UK); Anne-Genevieve Marcelin, Vincent Calvez (Université Pierre et Marie Curie, France); Maria Angeles Munoz (Servicio Madrileño de Salud-Hospital General Universitario Gregorio Marañon, Spain); Britta Wahren (Karolinska Institutet, Sweden); Caroline Foster (Imperial College Healthcare NHS Trust, London, UK); Mark Cotton (Stellenbosch University-Faculty of Medicine and Health Sciences, South Africa); Merlin Robb, Jintanat Ananworanich (The Henry M. Jackson Foundation for the Advancement of Military Medicine, Maryland); Polly Claiden (HIV i-Base, UK); Deenan Pillay (University of KwaZulu-Natal Africa Center, South Africa); Deborah Persaud (Johns Hopkins University); Rob J De Boer, Juliane Schröter, Anet J.N. Anelone (University of Utrecht, Netherlands); Thanyawee Puthanakit (Thai Red Cross AIDS-Research Centre, Thailand); Adriana Ceci, Viviana Giannuzzi (Consorzio per Valutazioni Biologiche e Farmacologiche, Italy); Kathrine Luzuriaga (University of Massachusetts Medical School, Worcester, Massachusetts); Nicolas Chomont (Centre de Recherche du Centre Hospitalier de l’Universitè de Montreal-University of Montreal, Canada); Mark Cameron (Case Western Reserve University, Cleveland, Ohio); Caterina Cancrini (Università degli Studi di Roma Tor Vergata, Italy); Andrew Yates, Louise Kuhn (Columbia University, New York); Avy Violari, Kennedy Otwombe (University of the Witwatersrand, Johannesburg [PHRU] South Africa); Ilaria Pepponi, Francesca Rocchi (Children's Hospital “Bambino Gesu”, Rome, Italy); Stefano Rinaldi (University of Miami, Miller School of Medicine, Florida); Alfredo Tagarro (Hospital 12 de Octubre, Universidad Complutense, Madrid, Spain); Maria Grazia Lain, Paula Vaz (Fundação Ariel Glaser contra o SIDA Pediátrico, Mozambique); Elisa Lopez, Tacita Nhampossa (Fundação Manhiça, Mozambique).

Author contributions: M.C. and R.G. performed the statistical analyses and drafted the article. A.B., A.J., D.G. and P.R. conceptualized and designed the study and, were involved in the preparation and review of the final article. All coauthors participated in discussions about interpretation of findings, were involved in the preparation and critical review of the final article. All participating cohorts within EPPICC were involved in the collection of data and interpretation of the findings.

This work was funded by the Early-treated Perinatally HIV-infected Individuals: Improving Children's Actual Life with Novel Immunotherapeutic Strategies (EPIICAL) consortium (http://www.epiical.org), supported by Paediatric European Network for Treatment of AIDS (PENTA) Foundation, funded through an independent grant by ViiV Healthcare UK. This work was also funded by Medical Research Council programme grant MC_UU_12023/26 awarded to the MRC Clinical Trials Unit and a pilot award to P.P. obtained by Children's Hospital Bambino Gesú (Ricerca corrente 2017 and 2018), and Associazione Volontari Bambino Gesù.

**Group authorship:** Man K. Chan^a^, Ruth Goodall^a^, Ali Judd^a^, Nigel Klein^b^, Elena Chiappini^c^, Thomas Klimkait^d^, Nicole Ngo-Giang-Huong^e^, Paolo Palma^f^, Paolo Rossi^f^, Claire Thorne^b^, Anna Turkova^a^, Paola Zangari^f^, Pieter L. Fraaij^g,h^, Dasja Pajkrt^i^, Laura Marques^j^, Intira J. Collins^a^, Diana M. Gibb^a^, Maria I. Gonzalez-Tome^k^, Maria L. Navarro^l^, Jose T. Ramos^m^, Antoni Noguera-Julian^n^, Josiane Warszawski^o^, Christoph Königs^p^, Vana Spoulou^q^, Filipa Prata^r^, Tessa Goetghebuer^s^, Luisa Galli^t^, Lars Naver^u^, Carlo Giaquinto^v^, Magdalena Marczynska^w^, Liubov Okhonskaia^x^, Ruslan Malyuta^y^, Alla Volokha^z^, Luminita Ene^aa^, Pablo Rojo^k^ and Abdel G.A. Babiker^a^

**Group authorship affiliations:**^a^MRC Clinical Trials Unit at UCL, Institute of Clinical Trials & Methodology, University College London (UCL), ^b^UCL Great Ormond Street Institute of Child Health, London, UK, ^c^University of Florence, Florence, Italy, ^d^University of Basel, Basel, Switzerland, ^e^MI 174 PHPT/Faculty of Medical Sciences, Chiang Mai University, Chiang Mai, Thailand, ^f^Research Unit in Congenital and Perinatal Infection, Academic Department of Pediatrics (DPUO), Children's Hospital Bambino Gesu‘, Rome, Italy, ^g^Department of Viroscience, Erasmus Medical Centre, ^h^Subdivision of Infectious Diseases and Immunology, Department of Pediatrics, Erasmus Medical Centre – Sophia, Rotterdam, ^i^Department of Pediatric Infectious Diseases, Emma Children's Hospital, Amsterdam UMC, University of Amsterdam, Amsterdam, The Netherlands, ^j^Paediatric Infectious Diseases and Immunodeficiencies Unit, Pediatric Department, Porto Central Hospital, Porto, Portugal, ^k^Peadiatric HIV and Infectious Diseases Department, Hospital Doce de Octubre, ^l^Pediatrics Infectious Diseases Unit, Hospital Universitario Gregorio, ^m^Paediatrics Department, Hospital Clínico Universitario San Carlos, Madrid, ^n^Unitat d’Infectologia, Servei de Pediatria, Hospital Sant Joan de Deu, Universitat de Barcelona, Barcelona, Spain, ^o^Institut National de la Santé et de la Recherche (INSERM), Paris, France, ^p^Department of Paediatrics, University Hospital Frankfurt, Goethe University, Frankfurt, Germany, ^q^Department of Infectious Diseases, University of Athens, Athens, Greece, ^r^Hospital de Santa Maria, Lisbon, Portugal, ^s^Hopital St Pierre, Brussels, Belgium, ^t^Universita Degli Studi Firenze, Firenze, Italy, ^u^Karolinska Institutet and University Hospital, Stockholm, Sweden, ^v^Paediatric European Network for the Treatment of AIDS (PENTA), Padova, Italy, ^w^Medical University of Warsaw, Hospital of Infectious Diseases, Warsaw, Poland, ^x^Republican Hospital of Infectious Diseases, St Petersburg, Russia, ^y^Perinatal Prevention of AIDS Initiative, Odessa, ^z^Shupyk National Medical Academy of Postgraduate Education, Kiev, Ukraine, and ^aa^Victor Babes Hospital, Bucharest, Romania.

### Conflicts of interest

There are no conflicts of interest.

## Supplementary Material

Supplemental Digital Content
